# From clinical management to personalized medicine: novel therapeutic approaches for ovarian clear cell cancer

**DOI:** 10.1186/s13048-024-01359-7

**Published:** 2024-02-12

**Authors:** Zesi Liu, Chunli Jing, Fandou Kong

**Affiliations:** 1 Department of Gynecology and Obstetrics, The First Affiliated Hospital of Dalian Medical University, Dalian, 116000 Liaoning Province China; 2 Department of Gynecology and Obstetrics, The Second Affiliated Hospital of Dalian Medical University, Dalian, 116000 Liaoning Province China

**Keywords:** Ovarian clear cell carcinoma, ARID1A, Immunotherapy, Targeted therapies, Synthetic lethality

## Abstract

Ovarian clear-cell cancer is a rare subtype of epithelial ovarian cancer with unique clinical and biological features. Despite optimal cytoreductive surgery and platinum-based chemotherapy being the standard of care, most patients experience drug resistance and a poor prognosis. Therefore, novel therapeutic approaches have been developed, including immune checkpoint blockade, angiogenesis-targeted therapy, ARID1A synthetic lethal interactions, targeting hepatocyte nuclear factor 1β, and ferroptosis. Refining predictive biomarkers can lead to more personalized medicine, identifying patients who would benefit from chemotherapy, targeted therapy, or immunotherapy. Collaboration between academic research groups is crucial for developing prognostic outcomes and conducting clinical trials to advance treatment for ovarian clear-cell cancer. Immediate progress is essential, and research efforts should prioritize the development of more effective therapeutic strategies to benefit all patients.

## Highlights

• Resistance to conventional chemotherapy is one of the main reasons for the worse prognosis of OCCC patients.

• FSS for OCCC patients with Stage IA and a strong desire to have children.

• VTE increases the risk of various adverse events in OCCC patients.

## Introduction

Epithelial ovarian cancer (EOC) stands as the prevailing form of ovarian cancer, which accounts for roughly 90% of all ovarian cancers (OC) [1]. Each year, there are approximately 239,000 new cases of EOC and 152,000 deaths worldwide [2, 3]. The World Health Organization (WHO) defines ovarian clear cell cancer (OCCC) as a malignant ovary tumor consisting of hyaline, eosinophilic and hobnail-like cells arranged in a tubulocystic, papillary, and solid structure. OCCC, as a specific pathological type of EOC, has a unique clinical presentation, biological behavior, histopathology, molecular features, and intrinsic chemoresistance. In addition, most patients with OCCC are younger than other subtypes of EOC at the time of diagnosis and are in the early stages [4, 5]. However, only 20–25% of patients with OCCC respond to conventional chemotherapeutic agents targeting EOC [6], and no effective strategy has been proposed for treating OCCC [7]. CA125 is the most commonly used diagnostic marker for OC, but its specificity and accuracy for OCCC diagnosis are poor [8]. As a result, advanced-stage OCCC patients have a poorer prognosis than advanced-stage EOC patients [4, 9].

By reviewing the relevant literature, this review summarizes the pathogenesis of OCCC and listed the possible mechanisms that may lead to chemoresistance. These may provide important new directions for future research on OCCC. It also explored and listed the latest therapeutic strategies to provide relevant clues for the clinical management of OCCC.

## Pathogenesis of OCCC

**Fig. 1:**
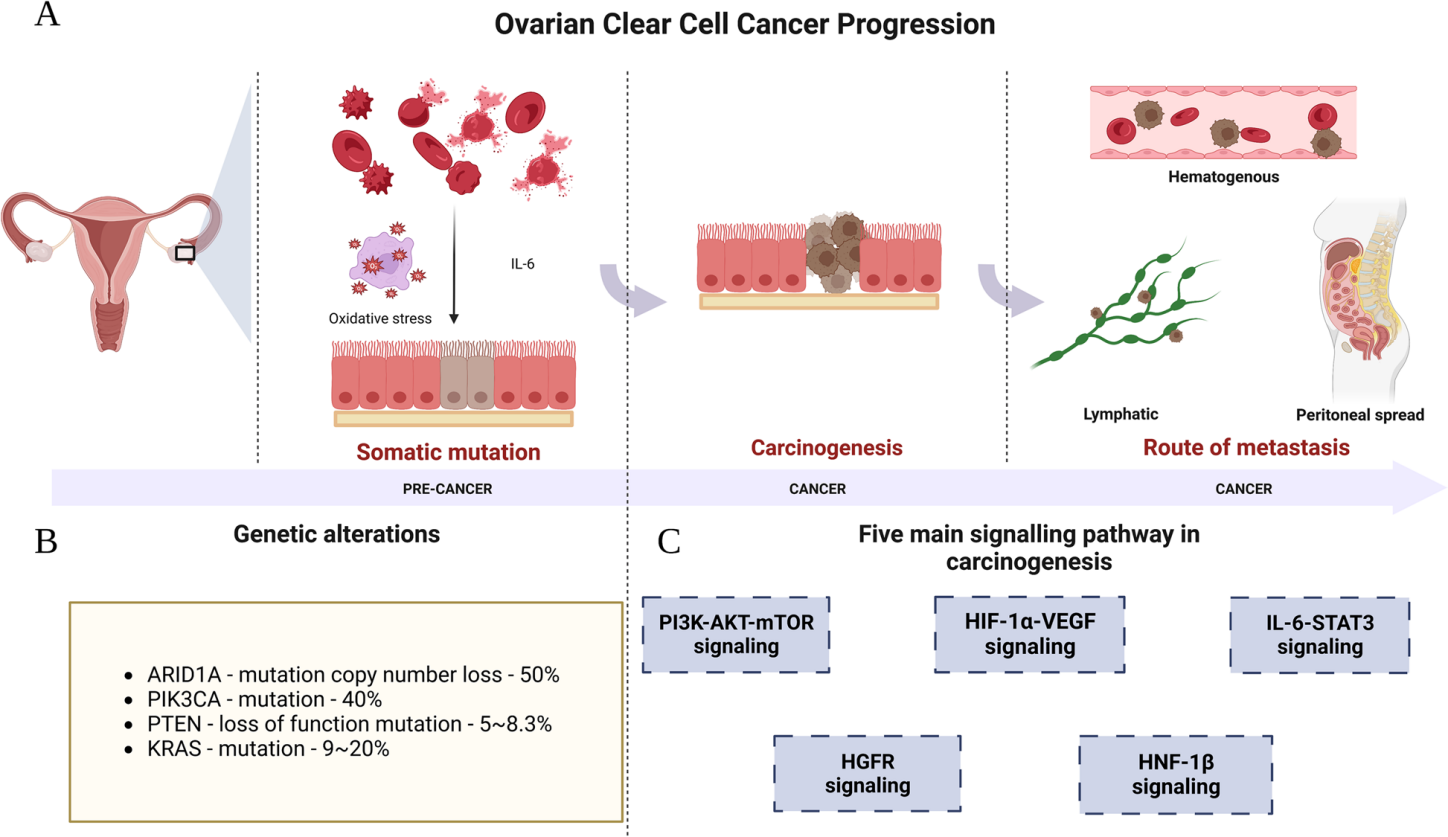
Schematic overview of ovarian clear cell cancer carcinogenesis. **A** Ovarian Clear Cell Cancer Progression; **B** Genetic alterations; **C** Five main signalling pathway in carcinogenesis. Shed menstrual endometrium leaves the cavity and retrograde along the fallopian tube to the ovary. The microenvironment of ectopic endometrial cells contains a large amount of fragmented erythrocytes which in turn releases a large amount of ions leading to stronger oxidative stress and more IL-6 production, stimulating cellular. At the same time, there is a higher mutational burden compared to normal endometrial cells, with common mutations including ARID1A, PIK3CA, PTEN, and KRAS. The five major pathways involved in carcinogenesis to OCCC include: PI3K-AKT-mTOR pathway, HIF-1α-VEGF pathway, IL-6-STAT3 pathway and MAPK pathway and HNF-1β pathway. In addition, lymphatic, hematogenous, and peritoneal implantation metastasis are common metastatic pathways in advanced ovarian clear cell carcinoma

Atypical EMs is widely considered precancerous lesion of OCCC. Several studies have shown that ovarian-implanted EMs retain the ability to proliferate, differentiate, and invade [10, 11]. As the menstrual cycle changes, the internal environment surrounding EMs cells shows a large amount of disrupted heme [12], which releases a large number of ions leading to stronger oxidative stress and more IL-6 production, stimulating cellular malignancy [13]. In an animal study, Chandler et al. found that IL-6 inflammatory cytokine signaling plays an important role in OCCC pathogenesis in mice (Fig. 1A) [14].

Ectopic endometrial cells have a higher genetic mutation burden than endometrial cells in the uterine cavity [15]. ARID1A mutation, BAF250 protein loss, and PIK3CA mutation are early molecular events in the development of OCCC [11]. A whole genome sequencing of OCCC suggested an average of 178 exon mutations and 343 somatic copy number variants per OCCC sample, with ARID1A (> 50%) and PIK3CA (> 40%) being the most commonly mutated genes (Fig. 1B) [16, 17]. The remaining key genetic alterations are shown in Tables 1, 2 and 3. A genetically engineered mouse model (GEM) study found that single activation of ARID1A alone could not lead to OCCC malignancy unless mutations in genes such as PTEN were induced [14]. In addition, Yamamoto et al. found that mutations in ARID1A are often accompanied by other critical genetic mutations, such as PIK3CA [18]. These results suggest that single gene mutation cannot cause cellular carcinogenesis, OCCC tumorigenesis may be accomplished by multiple genetic mutations in concert.

**Table 1:** Critical genetic changes in ovarian clear cell carcinoma

Gene	Frequency	Type of alteration	Original function/Pathway affected	Reference
ARID1A	40–57%	Mutation copy number loss	SWI-SNF chromatin remodeling complex DNA double-strand break (DSB) repair	[16, 19–22]
SMARCA4	5–18%	Mutation		[20, 23, 24]
ARID1B	6–18%	Mutation		[20, 25]
PI3KCA	> 50%	Mutation	PI3K-AKT pathway	[19, 26–28]
PTEN	5–8.3%	Loss-of-function mutation		[29, 30]
AKT2	8–26%	Amplification		[20, 22, 31]
ZNF217	20–36%	Amplification		[32–34]
KRAS	9–20%	Mutation	MAPK pathway	[19, 23, 34]
PPP2R1A	7–15%	Mutation		[19, 25, 35]
ERBB2	2–13%	Mutation and amplification		[22, 24, 26]
MET	24–37%	Amplification		[36, 37]
TP53	8.5–21.6%	Mutation	DNA repair	[24, 38, 39]
BRCA1/2	2.1–6%	Mutaion		[3, 40]
HNF-1β	> 90%	Hypo-methylation overexpression	Glucose metabolism	[13, 41, 42]

**Table 2:** Clinical trials of clear cell carcinoma exploiting ARID1A synthetic lethal interactions

Therapy strategy	Drug	Tumor type	Mechanism	Response summary	Reference
ATR-inhibitor	VX-970	OCCC	ARID1A deficiency results in topoisomerase 2A and cell cycle defects, which cause an increased reliance on ATR checkpoint activity. Therefore, inhibition of ATR triggers premature mitotic entry, genomic instability and apoptosis in ARID1A-loss tumor	In vitro studies, we found that the human OCCC cell line was more sensitive to VX-970 than the ARID1A wild-type OCCC cell line(*P* < 0.0001) and also exhibited a DNA damage and apoptotic response to ATR-inhibitor exposure. Moreover, in a mouse human OCCC transplantation model, the use of ATR inhibitors significantly inhibited the growth of ARID1A-loss tumors with no significant adverse effects on normal tissues	[43]
ATR-inhibitor + ICB therapy	AZD6738 Durvalumab	Advanced-stage and/or recurrent solid tumors	ART-inhibitors can disrupt DNA repair in tumor cells and thus lead to the release of more tumor cell antibodies	Objective response rate: 17% (2/12)	[44]
ATR-inhibitor + PARP-inhibitor	Ceralasertib Olaparib	OCCC	ARID1A is involved in the DNA double-strand brink process in tumor cells, similar to the role played by BRCA in tumor cells	On-going	[45]
AURKA-inhibitor	ENMD-2076	OCCC	AURKA acts as an oncogene that can cause tumor cell overriding cell cycle checkpoints by phosphorylate CDC25 Cat Ser198 via PLK1 and activate CDC25C nuclear translocation leading to tumor cells overriding cell cycle checkpoints, and enhancing cell proliferation, and suppressing apoptotic pathways. Loss of ARID1A can upregulate the expression level of AURKA	6- month PFS rate of ARID1A-loss OCCC patients: 33% 6- month PFS rate of ARID1A-wild OCCC patients: 12% *P* = 0.023	[46]
Tyrosine kinase inhibitor	Dasatinib	OCCC Endometrial CCC	Acting on P21 and Rb to cause more ARID1A-loss cells to enter the G1-S cell cycle arres Inhibiting the growth and division of cancer cells by inhibiting their angiogenesis	Response rate: 3.8% (1/28) Mean PFS: 2.14 months	[47]
EZH2 inhibitor	GSK126	OCCC	EZH2, as a key component of PCR2, modifies histone H3 on chromatin and wraps it tightly in nucleosomes, making chromatin more compact and thus suppressing gene transcription and expression Inhibition of EZH2 can lead to upregulation of PIK3IP1 expression, which in turn inhibits PI3K-AKT signaling and contributes to the synthetic lethal interaction between ARID1A and EZH2	ARID1A mutational status correlated with response to the EZH2 inhibitor The growth rate of ARID1A-loss OCCC cells: 28.4% The growth rate of ARID1A-wild OCCC cells: 66.0% *P* < 0.001	[48]
HDAC2 inhibitor	SAHA	OC	HDAC2 functions as a corepressor of EZH2 and a component of PRC2 to suppress the expression of EZH2/ARID1A target tumor suppressor genes such as PIK3IP1 to inhibit proliferation and promote apoptosis	Compared with ARID1A wild-type cells, the half maximal inhibitory concentration (IC50) of SAHA is significantly lower in ARID1A-mutated cells (*P* = 0.008) SAHA treatment significantly reduced the tumor burden (*P* < 0.001) and the amount of ascites formed (*P* = 0.009) in mice bearing ARID1A-mutated tumors	[49]
Glutamate-cysteine ligase synthetase catalytic subunit (GCLC) inhibitor	APR246 and PRIMA-1	OC	Controlling cysteine required for glutathione production and causes apoptosis triggered by excess reactive oxygen species	Compared with ARID1A wild-type cancer cells, glutathione is significantly reduced in ARID1A-deficient cancer cells and leads to massive apoptosis (*P* < 0.001)	[50]
HSF-1 inhibitor	NXP800 (Nuvectis)	OCCC	HSF1(Heat Shock Factor 1), as an ancient stress-inducible transcription factor, plays a key role in the transcriptional activation of the eukaryotic heat shock response and acts as a master transcriptional regulator of proteostasis. The HSF1 pathway has been shown to play a key role in oncogenesis and the hallmark features of malignancy and is important in the initiation and progression of many experimental cancer models	On-going	[51]
Bromodomain and extra terminal (BET) inhibitor	JQ1 iBET762	OCCC	BET inhibitors cause a reduction in the expression of multiple SWI/SNF members including ARID1B, providing a potential explanation for the observed lethal interaction with ARID1A loss	BET inhibitors can inhibit the proliferation of ARID1A mutant OCCC lines significantly (*P* < 0.001) ARID1A depletion enhances sensitivity to BET inhibitors (*P* < 0.001)	[52]

**Table 3:** Other molecular targeted therapies for ovarian clear cell cancer

Trial name/Clinical. gov. identifier	Phase	Target	Interventions	Patient type	Status
GOG-0268 (NCT01196429)	II	mTOR	Temsirolimus + Carboplatin + Paclitaxel	Stage III-IV OCCC	Completed
GOD-0254 (NCT00979992)	II	PDGFR VEGFR	Sunitinib	Persistent or recurrent OCCC	Completed
NRG-GY001 (NCT02315430)	II	MET RET VEGFR2 AXL	Cabozantinib	Persistent or recurrent OCCC	Completed
ENGOT-GYN1 (NCT02866370)	II	FGFR PDGFR VEGFR	Nintendanib	Persistent or recurrent OCCC and endometrial CCC	On-going
MEDI-4736 (NCT03405454)	II	PD-L1	Durvalumab	Persistent or recurrent OCCC	On-going
BrUOG354 (NCT03355976)	II	PD-L1 CTLA4	Nivolumab ± Ipilimumab	Persistent or recurrent OCCC or extra-renal origin CCC	On-going
NRG-GY016 (NCT03602586)	II	PD-1 IDO1	Pembrolozimab + Epacadostat	Persistent or recurrent OCCC	On-going
JGOG3022 (NCT04428398)	II	VEGF	Bevacizumab + Carboplatin + Paclitaxel	Stage III-IV epithelial ovarian cancer	Completed
JAVELIN Solid Tumor Trial (NCT01772004)	II	PD-L1	Avelumab	Persistent or recurrent OCCC	Completed

Mutated genes can regulate the expression of various proteins and the activity of signaling pathways to play an important role in the carcinogenesis of EMs cells. The main regulated proteins are involved in the five pathways: phosphatidylinositol 3-kinase (PI3K)/AKT/mammalian target of rapamycin (mTOR) pathway, hypoxia-inducible factor 1α (HIF-1α)/vascular endothelial growth factor (VEGF) pathway, hepatocyte nuclear factor 1β (HNF-1β) pathway, interleukin 6 (IL-6)/signal transducer and activator of transcription 3 (STAT3) pathway, and Hepatocyte growth factor receptor (HGFR) signaling pathway; they are involved in cell proliferation, chemoresistance, angiogenesis, and invasion (Fig. 1C) [36, 53–63]. The regulatory pathways mentioned above are potential targets for OCCC treatment in the future, which will be discussed in detail later.

Hsu et al. performed a comprehensive analysis using transcriptomic datasets from public domain databases to identify gene clusters that may be implicated in the pathogenesis of OCCC carcinogenesis [64]. Then, six functional gene clusters were identified and summarized: ribosomal protein, eukaryotic translation initiation factors, lactate, prostaglandin, proteasome, and insulin-like growth factor. The interactions between these six gene clusters build a network of OCCC pathogenesis.

## Chemoresistance

**Fig. 2:**
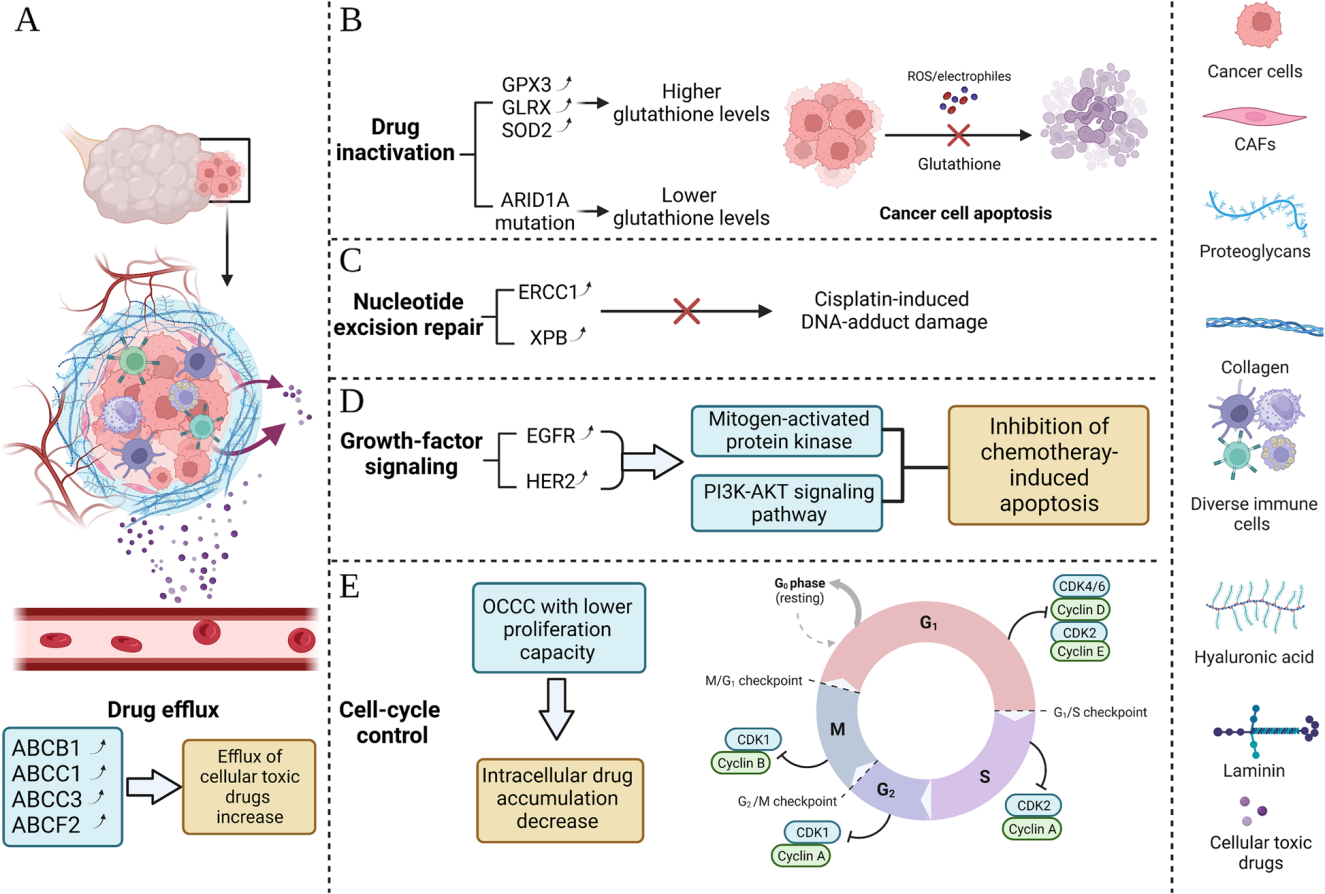
Recent-known mechanism of chemoresistance. There are five mechanisms thought to be involved in the lack of response of colorectal adenocarcinoma to pharmacological treatment, including drug efflux (**A**), drug inactivation (**B**), nucleotide excision repair (**C**), growth-factor signaling (**D**) and cell-cycle control (**E**)

The resistance of OCCC to conventional chemotherapy is the main reason for the poor prognosis of OCCC patients. Several recent studies have revealed the mechanisms associated with OCCC chemoresistance (Fig. 2).

### Drug efflux

Adenosine triphosphate-binding cassette subfamily B member 1 (ABCB1), Adenosine triphosphate-binding cassette subfamily C member 1 (ABCC1), Adenosine triphosphate-binding cassette subfamily C member 3 (ABCC3), Adenosine triphosphate-binding cassette subfamily transporter F2 (ABCF2), and members of the adenosine triphosphate binding cassette (ABC) can play an important role in OCCC chemoresistance by regulating intracellular drug concentrations [65]. ABCB1 and ABCC1 are ATP-dependent efflux pumps localized in the cell membrane and regulate the entry and exit of cellular toxic drugs, including paclitaxel (PTX) and carboplatin, into and out of cells [66, 67]. Itamochi et al. found that the expression levels of ABCB1 and ABCC1 correlated with the degree of resistance of OCCC cells to chemotherapeutic drugs [68]. In addition, Borst and colleagues found that ABCC3 modulates various substrates, including chemotherapeutic agents, which leads to chemoresistance in various malignancies [65]. By comparing the expression levels of ABCC3 in ovarian-serous adenocarcinoma (OSAC) and OCCC cells, Ohishi et al. found that the ABCC3 expression in OCCC cells was significantly higher than that in OSAC [69]. The results of Borst’s and Ohishi's study illustrated that ABCC3 might be involved in the chemoresistance of OCCC.

In addition, previous studies have revealed that NRF2 regulates cisplatin resistance in various malignancies, including ovarian cancer [70, 71]. ABCF2, as an NRF2 target gene, is positively correlated with NRF2 [71]. ABCF2-overexpressing cell lines containing high levels of NRF2 can reduce apoptosis and increase cell viability after cisplatin treatment, resulting in OCCC resistance to chemotherapy (Fig. 2A) [72, 73].

### Drug inactivation

It is well known that the intracellular concentration of antitumor drugs in tumor cells is the key point to improve the therapeutic effect. The glutathione (GSH) system plays a vital role in the metabolism of many chemotherapeutic agents and, therefore, in the chemoresistance of many malignancies [74]. Schwartz et al. found that some key genes in the GSH system, such as glutathione peroxidase 3 (GPX3), glutaredoxin (GLRX), and superoxide dismutase (SOD2), were expressed at higher levels in OCCC cells and were significantly increased when tumor cells were exposed to chemotherapeutic agents like platinum [68, 75]. Interestingly, ARID1A can affect SLC7A11 transcription and thus maintains GSH homeostasis. It was found that ARID1A-deficient OCCC cells are often accompanied by low expression of SLC7A11, resulting in low basal GSH levels and weakening the cancer cells' resistance to oxidative stress, leading to apoptosis (Fig. 2B) [19, 50].

### Nucleotide excision repair

Nucleotide excision repair is a multigene-regulated DNA damage repair pathway that contributes to drug resistance in tumor cells [76]. Excision repair cross-complementation group 1 (ERCC1) and xeroderma pigmentosum group B (XPB) as two key genes in the Nucleotide excision repair pathway were significantly upregulated in malignant cells were significantly upregulated (ERCC1: 0.76 vs. 0.09, *P* < 0.001; XPB: 0.91 vs. 0.04, *P* < 0.001). ERCC1 and XPB expressions were highest in OCCC among all EOC tissue subtypes, at 1.21 (*P* = 0.040) and 1.18 (*P* = 0.015), respectively [77]. On the one hand, ERCC1 expression and transcription in OCCC can activate AP1, a promoter activator, removing cisplatin-induced DNA-adduct damage and platinum resistance and treatment failure [78, 79]. On the other hand, ERCC1 and XPB were found to be upregulated after cisplatin treatment [80, 81]. hMLH1 and hMSH2 are important components of the DNA mismatch repair system (MMR) [82]. Various studies have found a strong correlation between altered expression of hMLH1 and hMSH2 in CCC tumors and microsatellite instability (MSI). Moreover, hMLH1 and hMSH2 expressions were significantly upregulated during the malignant transformation of OCCC [83, 84]. The above results suggest that nucleotide excision repair may be involved in the chemotherapeutic drug resistance of OCCC (Fig. 2C).

### Growth-factor signaling

Epidermal growth factor receptor (EGFR) and v-erb-b2 erythroblastic leukemia viral oncogene homolog 2 (HER2) phosphorylate Bc1-2 antagonist of cell death (BAD) and B-cell leukemia/ lymphoma (Bcl)-2 by regulating mitogen-activated protein kinase and phosphatidylinositol 3′-kinase (PI3K) –AKT signaling pathways and inhibiting of chemotherapy-induced apoptosis [85–87]. Several studies have demonstrated the involvement of EGFR and HER2 in OC chemoresistance and their association with poor prognostic outcomes in ovarian cancer patients [88–90]. An immunohistochemical study indicated that upregulation of EGFR expression could be found in 61% of OCCC cells [91]. An animal study further revealed the potential mechanism of EGFR involvement in chemoresistance. Specifically, in nude mice, silencing fibroblast growth factor receptor 3 (FGFR3) blocked EGFR phosphorylation and thus suppressed the activation of the PI3K/AKT pathway, thereby improving platinum-based chemotherapy's efficacy [92]. On the other hand, as a proto-oncogene, HER2 encodes a transmembrane tyrosine kinase receptor of the epidermal growth factor family. The overexpression of HER2 can be detected in 20 to 25% of ovarian cancer cases [93]. In addition, a study of OCCC noted that molecular changes in HER2 were significantly associated with papillary dominant growth patterns (*p* = 0.005) [94]. Therefore, HER2 status is not only a predictive biomarker for the therapeutic response to anti-HER2 therapies in various but also a signal that indicates a poor sensitivity to conventional anticancer agents (Fig. 2D).

### Cell-cycle control

Cytotoxic drugs are primarily effective against proliferating cells [95]. However, intracellular drug accumulation in resting cells decreases, leading to chemotherapy drug resistance [96, 97]. Itamochi et al. found that OCCC had a longer doubling time compared to high-grade serous ovarian carcinoma (HGSOC) (61.4 h vs. 29.8 h) [98] and when chemoresistant cells were derived from OCCC cell lines, Kusumoto et al. observed that the expression level of inhibin-α was elevated [99], those findings suggested that the chemoresistance of OCCC cells might be due to their less proliferative nature. Although it cannot be ruled out that this result is due to the high prevalence of stage I in OCCC patients, the chemoresistance in OCCC may be linked to its low proliferation. The cell cycle is controlled by cyclin-dependent kinases (CDKs), which are regulated by cyclin binding, phosphorylation, and CDK inhibitors [100]. Therefore, the researchers investigated the cell cycle regulatory molecules in OCCC and found that the expression of Ki-67 and cell cycle protein A were downregulated while the expression of p21 and cell cycle protein E significantly increased in OCCC cells [101, 102]. Ki-67 is expressed in all states of the cell cycle as an a-nuclear antigen, except for the resting cells of G0 [103, 104], Its expression in OCCC cells was significantly lower than in other types of EOC, and higher Ki-67 expression was found only in OCCC cells that responded to chemotherapy [105]. This finding suggests that targeting Ki-67 may increase the concentration of antitumor drugs in cells by controlling the tumor cell cycle in OCCC patients (Fig. 2E).

## Standard of therapeutic strategy

**Fig. 3:**
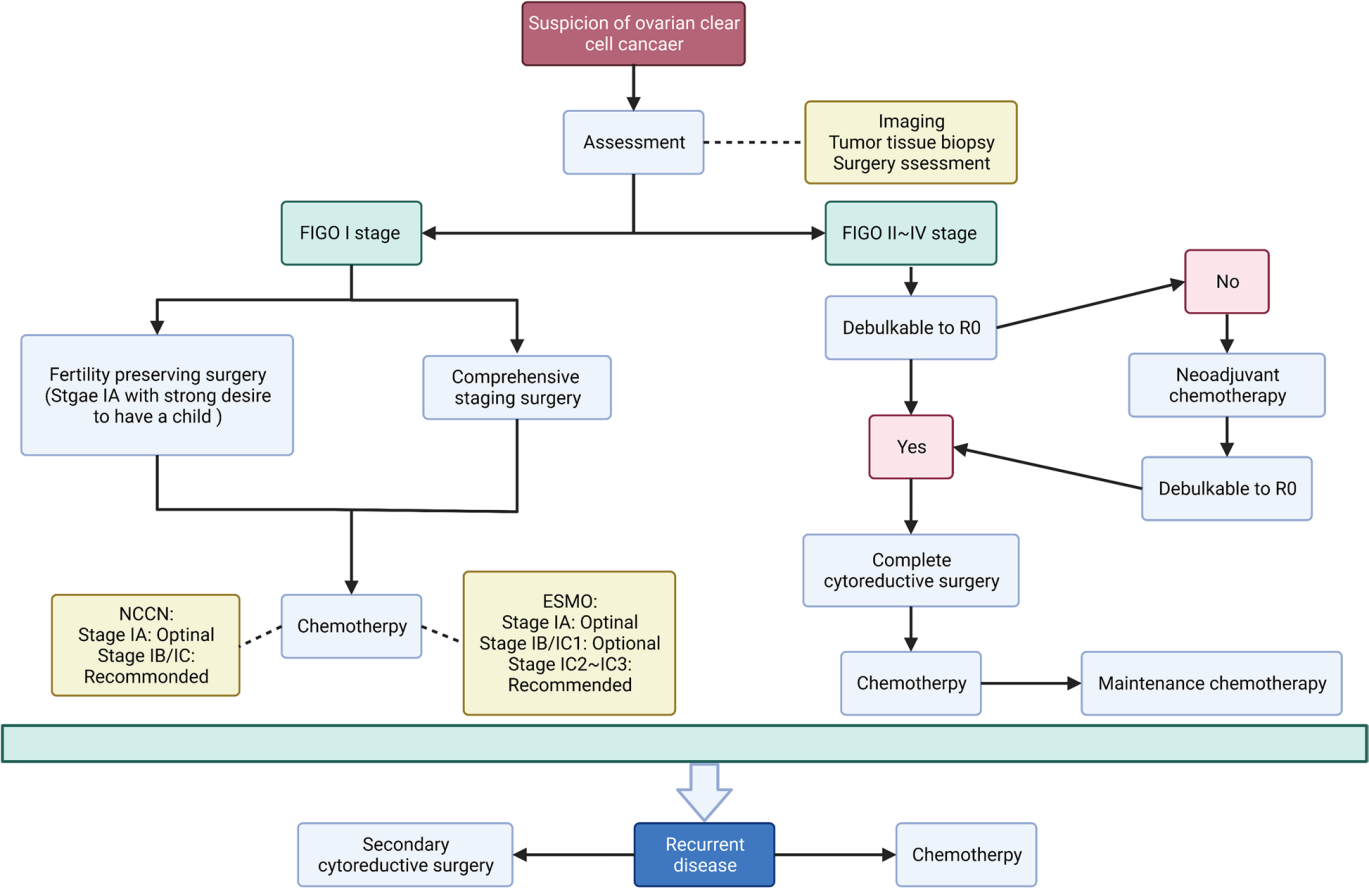
Ovarian clear cell cancer treatment strategies. Abbreviation: FIGO, Federation International of Gynecology and Obstetrics; NCCN, National Comprehensive Cancer Network; ESMO, European Society for Medical Oncology-European Society of Gynaecological Oncology

According to the National Comprehensive Cancer Network (NCCN) clinical practice guidelines, the treatment strategy for OCCC is the same as for EOC, i.e., full-stage surgery or tumor reduction with platinum-based systemic chemotherapy [106, 107]. However, due to the unique histological type and biology of OCCC, the RR to current first- and second-line chemotherapy regimens for EOC is lower for OCCC patients (Fig. 3).

### FIGO I Stage

#### Surgery

Comprehensive staging surgery is recommended for all Federation International of Gynecology and Obstetrics (FIGO) I stage OCCC patients, with the standard procedure being: bilateral salpingo-oophorectomy with total hysterectomy, peritoneal washing, peritoneal biopsies, omentectomy, and bilateral pelvic and para-aortic lymphadenectomy. Although studies have been published on the use of laparoscopy for extensive staging surgery of ovarian cancer, open surgery is currently recommended [108, 109]. Any suspicious lesions should be fully resected during surgery to achieve R0 and improve the patient's prognosis [110]. Since most OCCC patients are combined with EMs and may have severe pelvic adhesions, they should be carefully operated on to avoid accidental injury to other organs. Furthermore, the principle of being tumor-free should be followed to the greatest extent possible to avoid tumor rupture due to medical factors. If a tumor rupture occurs before surgery, a large amount of fluid (saline or distilled water) should be used to flush the abdominal cavity to avoid tumor cell dissemination and implantation [106, 107]. The impact of tumor rupture on patient prognosis is discussed below in detail. Although the percentage of positive ascites cytology is lower in patients with stage I, positive ascites cytology in early OCCC patients implies a higher percentage of lymph node (LN) metastases (10.2% vs. 2.8%) and ovarian surface involvement (11.8% vs. 2.8%) [111]. Therefore, a thorough intraoperative tumor cytology examination is essential. The Gynecologic Cancer InterGroup (GCIG) clearly states that systemic LN dissection can help define the stage of OCCC patients, predict the prognosis and guide the subsequent treatment, but it is unsure whether systemic lymph node dissection should be performed in early-stage OCCC patients [112]. Mahbi et al. showed that the degree of LN stripping was associated with disease-specific survival (DSS) in patients with OCCC [113]. Also, the number of LNs resected is a potential prognostic predictor of early OCCC, with a higher number of LNs resected implying longer progression-free survival (PFS) (cut-off value: 35) [114]. On the other hand, as the number of resection lymph nodes (RLNs) increases, the risk of postoperative complications such as infection and lymphatic leakage increases, thus affecting patient prognosis [115]. In addition, the NCCN study found that the rate of LN metastasis in early-stage OCCC patients was only 15% [106]. Similarly, Heitz et al. showed that the probability of LN metastasis in early OCCC is relatively low, with only 3.6% in pT1aM0 and pT2aM0, compared with 71.6% in HGSOC [116]. Neither Suzuki nor Magazzino's study found a significant difference in overall survival (OS) between early-stage OCCC patients who underwent systemic LN dissection and those who did not [117, 118]. Therefore, in order to more effectively determine the status of LNs in OCCC patients, various novel LN staging systems have been proposed in recent years with good predictive performance, which will be further elaborated on in later sections.

There is still debate about whether early OCCC patients should undergo fertility-preserving surgery (FSS), which includes unilateral salpingo-oophorectomy and comprehensive surgical staging. Although the ESMO-ESGO guidelines do not currently recommend FSS for young OCCC patients [107], several studies have found that FSS does not have a negative impact on the prognosis of OCCC patients. A Korean study compared the prognosis of 22 patients with early OCCC who underwent FSS and 25 patients who underwent full-stage surgery; it found no significant differences in 5-year OC (*p* = 0.935), 5-year DFS (*p* = 0.849), and time to recurrence (*p* = 0.840) between the two groups [119]. Similarly, a study from the National Cancer Database (NCDB), after correcting for the performance of lymphadenectomy and disease substage, found that FSS was not an independent risk factor for the prognosis of patients with OCCC (HR. 0.83, 95% CI: 0.30 to 2.32). Furthermore, researchers conducted a systematic review of the literature and identified 132 patients with stage I disease who underwent FSS and discovered that only 15.2% (20/132) survived a median of 18 months after surgery. This result suggests that FSS does not increase the probability of recurrence in patients with OCCC. On the other hand, 5-year PFS and OS were 100% for 15 patients with stage IA FSS (60% of whom received platinum-containing combination chemotherapy) and only 66.0% and 93.3% for 15 patients with stage IC FSS (73.3% of whom received platinum-containing combination chemotherapy) [120]. Based on the evidence from the above studies, we believe that FSS is only indicated for patients with stage IA OCCC who strongly desire to have children and are under close follow-up conditions.

#### Chemotherapy

There is controversy about whether patients with stage I OCCC should be treated with chemotherapy and the choice and duration of chemotherapy agents. On the one hand, several studies have found that adjuvant chemotherapy does not improve the 5-year recurrence-free survival of stage IA/C OCCC patients (IA stage: 100% vs. 93%; IC stage: 94%) and survival rate of stage IA/C OCCC (IA stage: 100% vs. 93.8%; IC stage: 94.1% vs 86.6%) [121]. In order to investigate the effect of adjuvant chemotherapy on early OCCC, a study was conducted by Japanese scholars that included 219 patients with I-stage OCCC, of whom 195 patients received adjuvant chemotherapy, and 24 did not. The results suggested that adjuvant chemotherapy was not an independent risk factor for the prognosis of patients with stage I OCCC (HR: 1.30 95% CI: 0.16–10.4) [122]. Similarly, by comparing the prognosis of 30 stage-I patients receiving adjuvant chemotherapy with 43 stage-I patients not receiving adjuvant chemotherapy, the investigators found no significant differences in 5-year PFS and 5-year OS rates between the two groups (PFS: 69. 6%-80.1% vs. 34.6%-73.9%; OS: 75.0%-87.4% vs. 70.0%-82.7%). After correcting for confounders such as age, substage, and year of diagnosis for both the EOC and OCCC, adjuvant chemotherapy remained unrelated to OS improvement in stage IA OCCC (HR: 1. 013; 95% CI: 0.802–1.281). However, adjuvant chemotherapy was a protective factor for patients in stage IC (HR: 0.583; 95% CI: 0.359–0.949) [123, 124]. In contrast, a study from the National Cancer Database found that patients with OCCC who received postoperative adjuvant chemotherapy had better prognostic outcomes than those who did not receive chemotherapy (89.2% vs. 86.2%, *P* < 0.001) [125]. The NCCN guidelines recommend the combination of PTX + single-agent carboplatin (TC) for all patients with stage I OCCC, while the ESMO considers TC alone to be effective and recommends postoperative chemotherapy for IC2 and IC3 stage OCCC patients only [106, 107].

Unlike HGSOC, researchers found no statistically significant difference in the risk of recurrence in patients with early-stage OCCC who received six cycles of chemotherapy versus three cycles of chemotherapy in a preliminary clinical trial (HR = 0.94, 95% CI = 0.60–1.49) [110]. In a Japanese study that included 5 institutions with early-stage OCCC, 38 patients (18.1%) received 3 cycles, and 172 (81.9%) received 6 cycles of adjuvant treatment and were divided into two groups. A comparison of PFS, OS, and recurrence rates between the two groups showed that postoperative adjuvant chemotherapy was not associated with recurrence rate (18.4% vs. 27.3%, *P* = 0.40) and it did not improve the survival time (PFS: HR: 1.4; 95% CI: 0.63–3.12; OS: HR 1.65; 95% CI: 0.59–4.65) [126].

Based on the above results, we believe that the benefit of postoperative adjuvant chemotherapy in patients with stage IA OCCC is uncertain, with a better prognosis for stage IA patients. Stage IA patients who are fully informed and have close follow-ups can choose whether to have adjuvant chemotherapy or observation according to their circumstances.

### FIGO II-IV stage

#### Surgery

Tumor cell reduction combined with TC protocol chemotherapy is currently the standard treatment strategy for all EOC, including OCCC. As OCCC has a low RR of 11%-50% to chemotherapy, unlike other EOC subtypes, the ability to perform satisfactory tumor reduction becomes a key factor affecting the prognosis of patients with advanced-stage OCCC [6, 9]. Several studies have shown that minimizing the extent of postoperative residual lesions to achieve no residual visualization (R0) significantly prolongs patient survival, especially for advanced-stage OCCC patients. A study in Taiwan that included 891 EOC patients, with 169 OCCC patients, found that residual malignancies were independent prognostic factors for cancer-specific survival (CSS). Specifically, 5-year CSS and 5-year survival after recurrence (SAR) were 82.3% (*P* = 0.010) and 14.3% (*P* = 0.002), respectively, in OCCC patients after satisfactory reduction, which was significantly better than other EOC subtypies [127]. These findings were further supported by a clinical trial that included 126 patients with II-IV stage OCCC, in which researchers found that chemotherapy did not affect the prognosis of patients with advanced-stage OCCC (HR: 0.56; 95% CI: 0.48–1.88), while residual tumor diameter (cut-off value: > 1 cm) was the only independent risk factor associated with prognosis (HR: 3.17; 95% CI: 1.68–6.00). (Off value: > 1 cm) was the only independent risk factor associated with prognosis (HR: 3.17; 95% CI: 1.68–6.00) [109].

#### NACT and chemotherapy

Because most EOCs are characterized by peritoneal disseminated metastases, neoadjuvant chemotherapy (NACT) can be considered for advanced-stage EOC to reduce tumor volume, decrease surgical difficulty, and improve surgical success. However, patients with OCCC do not seem to benefit from NACT due to the chemoresistance of OCCC. Results from a clinical trial (EORTC55971) of stage IIIC and IV OCCC suggest that the hazard ratio (HR) for death was 0.98 (90% CI: 0.84–1.13), and HR for the progressive disease was 1.01 (90% CI, 0.89–1.15) [128]. Similarly, a randomized controlled trial (CHORUS) revealed that patients treated with NACT + cytoreductive surgery did not improve prognostic outcomes compared to PDS, and patients had more severe chemotherapy-related side effects [129].

Traditional TC regimens have played a crucial role in improving the prognosis of HGSOC patients. However, only a small proportion of platinum-sensitive OCCC patients can benefit from TC regimens. Therefore, other novel chemotherapeutic regimens have been gradually introduced into the clinic. In a study that included 20 patients with recurrent or persistent OCCC, researchers found that gemcitabine monotherapy showed the best RR (60%, 1 for partial response and 2 stable diseases) and better prognostic outcome (median survival = 18 months). Only one patient treated with docetaxel plus irinotecan (9%) showed partial response. Stable disease was observed in 1 of 9 cases on a paclitaxel/carboplatin doublet and in 1 case on a docetaxel/carboplatin doublet [130]. In addition, two in vitro studies found that OCCC with ARID1A-loss was more likely to benefit from gemcitabine treatment, with 22% and 60% RR, respectively [130, 131]. Since an in vitro trial in 2002 showed that irinotecan (CPT-11) might be an effective treatment for OCCC patients, the use of CPT-11 plus cisplatin (CPT-P) therapy for the treatment of OCCC patients has become a hot topic of research and the results of several case reports and small phase II clinical trials have shown that CPT-P is effective in improving the prognosis of OCCC patients [132–134]. However, an extensive multicenter phase III clinical study conducted by the Japanese Oncology Society in 2016 overturned these findings. The study included 619 OCCC patients and randomized them to the CPT + P group (314 patients, CPT 60 mg/m2, days 1, 8, and 15; DDP 60 mg/m2, day 1; once every 4 weeks for 6 cycles) versus the TC group (305 patients, paclitaxel 175 mg/m2, carboplatin AUC6, once every 3 weeks for 6 cycles), resulting in no statistically significant differences in 2-year PFS rate (73% vs. 77.6%) and OS rate (85.5% vs. 87.4%) between the two groups. The study concluded that CPT-P was not superior to the standard TC regimen. In addition, the CPT + P group was more likely to have severe complications such as nausea, diarrhea, vomiting, and neutropenic fever predominantly [135].

### Recurrent disease

#### Secondary cytoreductive surgery

A study that included 61 patients with OCCC found that the prognostic outcome of patients with recurrent OCCC was related to the number of recurrent lesions and the site of recurrence. Compared to patients with nodal recurrence (30.1 months) and patients with multiple-site recurrences (13.7 months), patients with single-site recurrence had a longer median post-relapse overall survival (54.4 months) (*p* = 0.0002) [136]. Although the secondary reduction in patients with platinum-sensitive other subtypes recurrent EOC significantly prolonged patient PFS (14.0 months vs. 19.6 months; *P* < 0.001) and a benefit in the time to the start of first subsequent therapy of 7.1 months (13.9 months vs. 21 months; *P* < 0.001), patients with recurrent OCCC do not appear to benefit from the secondary reduction, which was found in 50% of patients who underwent secondary reduction after a disease-free interval of more than 30 months after relapse) [137]. The results of Kajiyama's study similarly indicate no difference in post-recurrence survival between the second and non-second surgery groups (21.2 months vs. 15.7 months, *P* = 0.318). Although patients who underwent secondary reduction had more prolonged post-recurrence survival after excluding patients who did not undergo satisfactory reduction, the difference between the two groups was not statistically significant (30.1 months vs. 15.7 months, *P* = 0.114) [138]. Based on those results, second cytoreductive surgery would not be a good choice for patients with recurrent OCCC.

#### Chemotherapy

Second-line chemotherapy for most relapsed EOC is an effective way to prolong patient survival. After recurrence, platinum-based chemotherapy is generally recommended for platinum-sensitive patients [107]. However, Takano noted that the RR for second-line chemotherapy after relapse was much lower than for other EOCs, with a rate of only about 10%, even in platinum-sensitive OCCC with initial chemotherapy [6]. In addition, a MD Anderson Cancer Center study found that the median PFS time for patients with relapsed OCCC was 8 months (95% CI: 4.6- 11.6 months). The median OS was 18 months (95% CI: 12.1–24.2 months). Among platinum-sensitive patients with relapsed OCCC who received platinum-based second-line chemotherapy, 2 patients (9%) had partial responses to retreatment with the TC regimen, and 4 (18%) had stable disease, while only 1% of platinum-resistant patients had partial responses to gemcitabine. Only 1% of platinum-resistant patients had partial responses to gemcitabine, and another 1% had stable disease after treatment with PTX and gemcitabine [139]. The current palliative treatment of choice for patients with platinum-resistant OCCC is non-platinum monotherapy, including weekly PTX, liposomal doxorubicin, gemcitabine, or topotecan, which can be combined with bevacizumab for maintenance therapy [140]. However, only a few small trials have shown that these therapies are effective, and there is still a lack of multicenter, large-scale, randomized controlled trials to confirm efficacy.

## Novel therapeutic strategies

### Immune checkpoint blockade

Immune checkpoint blockade (ICB) therapy is an innovative approach to treating malignant tumors by activating the patient's immune system to attack cancer cells, improving prognosis and prolonging survival. Many clinical studies with ICB therapy for OC have yielded promising results [141, 142]. Recent studies have found that the expression levels of genes related to inflammation and immune response, such as Interleukin-8 (IL-8), STAT3, Nuclear Factor kappa B (NF-κB), and Toll-like receptor-4 (TLR4), are significantly elevated in OCCC, suggesting that the development of OCCC may be linked with the microenvironment of immune suppression in patients [143, 144]. Meanwhile, with the development of bioinformatics and gene sequencing technologies, several studies have found abnormal expression of various immune checkpoint genes in OCCC, including CTLA-4, PD-1, PD-L1, LAG3, and Tim3, which provide potential targets for ICB therapy for OCCC [145]. These results all suggested that OCCC patients may benefit from ICB treatment and that some clinical trials have been conducted. The JAVELIN Solid Tumor Trial enrolled 125 patients with advanced OC and treated with Avelumab, of whom 2 patients with recurrent or refractory OCCC patients achieved a partial response (PR) (one of which proved to be immune-related PR) [146]. The phase II study of pembrolizumab (NCT02674061) included 19 patients with recurrent OCCC, and although the RR for treatment with pembrolizumab was only 15.8%, adverse events were well tolerated, suggesting that the use of pembrolizumab does not affect the quality of life of OCCC patients [147]. Nivolumab, an anti-PD-1 antibody, was used by Hamanishi et al. to treat patients with platinum-resistant OC [148]. They found a disease control rate of 45% in all 20 patients with a median PFS time of 3.5 months and the median OS time was 20.0 months, and only 2 patients had severe adverse events. One of the two OCCC patients achieved a complete response (CR), while the median OS time was 20.0 months. In addition, a multicenter phase II randomized trial of durvalumab (MEDI-4736) is currently underway to compare the efficacy of standard chemotherapy with PD-1/PD-L1 blockade therapy [149]. Results will be reported in the near future.

The major mismatch repair genes include MutL Homolog 1 (MLH1), MutS Homolog 2 (MSH2), MutS homolog 6 (MSH6), and Postmeiotic segregation increased 2 (PMS2), mutations in any one of which result in MMR defects [150]. The prevalence of MMR defects in OCCC is as high as 67% [151]. Previous studies found that ARID1A recruits the MSH2 to chromatin and is thus involved in the MMR of OCCC [152]. Li et al. found that ARID1A mutations were associated with increased immune activity in gastrointestinal cancer by causing MSI, and increased tumor mutational burden (TMB), producing more tumor-associated antigens and, thus, promoting anti-tumor immunity [153]. In addition, an in vitro trial found higher tumor-infiltrating lymphocytes and PD-L1 expression in the ARID1A-deficient OC cell line [152]. These results provide a new direction for the future treatment of OCCC, i.e., key gene targeting therapy combined with ICB therapy may improve the therapeutic effect of monotherapy.

Meanwhile, other immune checkpoint genes also deserve further investigation, such as cytotoxic T lymphocyte-associated protein 4 (CTLA4), expressed on regulatory T cells and activated cytotoxic T cells, which can suppress the function of T cells and the immune system's attack on tumors [154, 155]. At the same time, many studies have shown that CTLA-4 inhibitors can also increase the activity of cytotoxic T cells and achieve satisfactory results in tumors such as melanoma and small-cell lung cancer [156, 157]. A phase II clinical study of recurrent OCCC with the combination of PD-1 inhibitor (Pembrolizumab) and indoleamine-pyrrole 2,3-dioxygenase (IDO) inhibitor (Epacadostat) discovered that patients had a 21% overall RR, with a mean PFS and OS of 4.8 and 18.9 months, respectively. Recently, tumor vaccines are becoming a hot topic in tumor immunotherapy, and the primary tumor vaccines carried out against OCCC are Glypcan-3 (GPC3), a member of the Glypcan family acetyl heparan sulfate protein glycoprotein family, which is overexpressed in OCCC [145, 158]. However, only two small studies have included 66 and 32 OCCC patients treated with GPC3-derived peptide vaccines, and the results were not satisfactory, with an overall RR of 9.4% (2 PRs and 1 stable disease) [159] and a disease control rate of 17.9% [160].

**Fig. 4:**
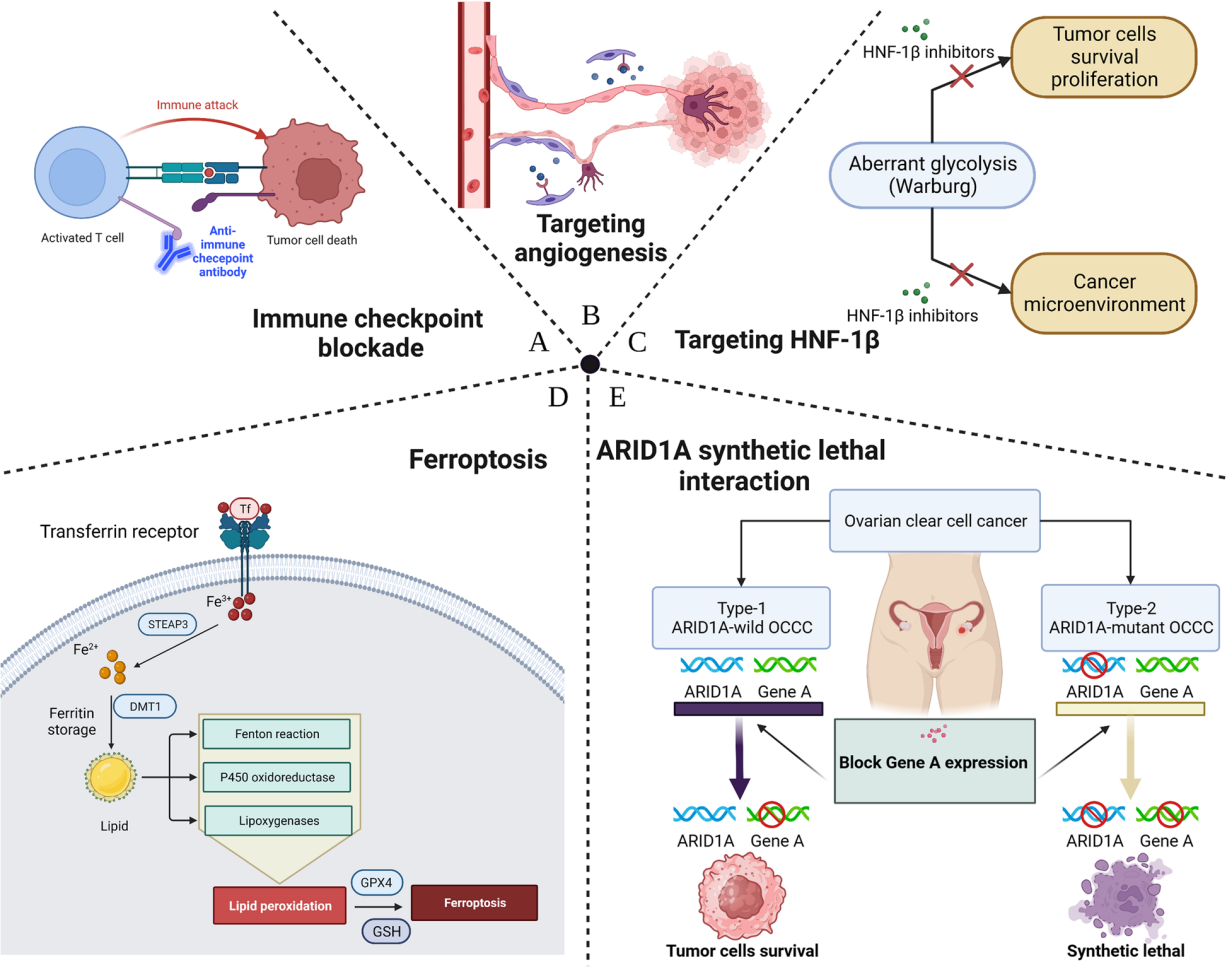
Recent-known novel therapies for ovarian clear cell cancer. Five major mechanisms of potential therapeutic target have been characterized in ovarian clear cell carcinoma, including immune checkpoint blockade (**A**), targeting angiogenesis (**B**), targeting HNF-1β (**C**), ferroptosis (**D**) and ARID1A synthetic lethal interaction (**E**)

The current ICB therapies for OCCC have small sample sizes and are unsatisfactory. Therefore, future multicenter, large-sample clinical trials are needed to further validate the efficacy of ICB for OCCC patients. Key gene targeting therapy combined with ICB therapy also should be considered as an efficient strategy for OCCC in future (Fig. 4A).

### Targeting angiogenesis

The generation of new vascular structures occurs throughout the whole process of development and progression of malignant tumors, including migration, proliferation, and differentiation of vascular endothelial cells. The new angiogenesis can help malignant cells obtain sufficient nutrients and oxygen to support their growth, invasion, and metastasis, becoming a hall marker of malignant tumors [161, 162]. Previous findings suggested that various angiogenesis-related cytokines and signaling pathways, such as vascular endothelial growth factor (VEGF) and IL-6/STAT/HF1 pathways, were involved in the development of OCCC and are closely related to the prognosis of OCCC patients [41]. For example, in the case of VEGF, activation of VEGF was found in 90% of OCCC patients and proved to be involved in the chemoresistance of OCCC cells, implying a poor prognosis [163]. In a prospective observational study (JGOG3022), investigators used bevacizumab in combination with platinum-taxane chemotherapy as a treatment strategy for advanced OCCC. They found that OCCC patients had a high RR to chemotherapy of 63.3%, a median platinum-free interval of 11.5 months, and all adverse events were tolerable [164]. These results suggest that anti-angiogenic drugs may improve the sensitivity of OCCC patients to chemotherapy and improve the prognostic outcome. However, the efficacy of bevacizumab as a maintenance drug following chemotherapy in OCCC patients is unknown. In the ROSiA Single-Arm Phase 3B Study (NCT01239732), after debulking surgery, OCCC patients received bevacizumab 15 (or 7.5) mg/kg every 3 weeks (q3w) with 4 to 8 cycles of paclitaxel (investigator’s choice of 175 mg/m2 q3w or 80 mg/m2 weekly) plus carboplatin AUC 5 to 6 q3w. Single-agent bevacizumab was continued until progression or for up to 24 months. However, the study findings revealed that OCCC patients do not benefit as much from bevacizumab maintenance treatment as other EOC subtypes [165].

On the other hand, Nintedanib is a multi-targeted tyrosine kinase inhibitor used to treat various malignancies, such as non-small cell lung cancer and OC. It inhibits the signaling pathways of various cytokines and their receptors, including vascular endothelial growth factor receptor, platelet-derived growth factor receptor, and fibroblast growth factor receptor, thereby inhibiting the proliferation, migration, and angiogenesis of tumor cells for the treatment of cancer [166, 167]. A randomized phase II study compared the efficacy and safety of nintedanib (BIBF1120) with standard chemotherapy in patients with recurrent OCCC (NICCC/ENGOT-OV36). Although there was no significant difference in PFS (2.3 months vs. 1.9 months) between the two groups, patients receiving nintedanib had higher 6- and 12-month-OS rates (19.7% vs. 8.9%), RR (2.1% vs. 0%), and disease control rate (DCR) (1.1% vs. 0%). rate (DCR) (16 weeks; 23.4% vs. 9.1%). In addition, Lheureux et al. performed an oral ENMD-2076 treatment for OCCC and found that ARID1A-deficient OCCC patients had a higher 6-month PFS rate than ARID1A-positive patients (33% vs. 12%), which implies that ARID1A may be a potential predictive biomarker for anti-angiogenic therapy in OCCC patients [46]. Although the results of the current anti-angiogenic clinical trials are not satisfactory, the Lheureux study suggests that researchers should pay more attention to finding potentially effective biomarkers to screen for OCCC patients who may benefit from anti-angiogenic therapy in future studies. Also, more studies should be conducted on combining anti-angiogenic drugs with other agents to treat OCCC patients. For example, a phase I clinical trial used immune checkpoint blockade therapy (durvalumab) in combination with an antiangiogenic agent (cediranib) to treat a patient with recurrent OCCC and achieved a partial response rate [168]. A phase III IMagyn050/GOG 3015/ENGOT-OV39 trial reported a numerical increase in PFS by adding atezolizumab in non-high-grade serous histology, including clear cells (Fig. 4B) [169].

### Targeting hepatocyte nuclear factor‑1β

Because the tumor microenvironment is frequently hypoxic, tumor cells produce ATP for proliferation and invasion via lactic acid fermentation in the glycolytic pathway rather than the normal oxidative phosphorylation pathway, which is known as the Warburg effect [170]. This effect is also observed in the OCCC [42]. Hepatocyte nuclear factor-1β (HNF-1β) is a transcription factor belonging to the HNF family, which plays an essential role in embryonic development and adult physiology, mainly involved in regulating the development and function of organs such as the liver, pancreas, intestine, and urinary system. In recent years has been gradually considered to be involved in regulating the development of various tumors, including OCCC [171]. HNF-1β can play an essential role in the Warburg effect of tumors by converting the glucose metabolism of tumor cells from oxidative phosphorylation to glycolysis, thereby producing more lactic acid and reducing citric acid [172]. Long-term exposure to oxidative stress is a potential mechanism for the malignant transformation of EMs into OCCC, as mentioned before. Previous studies found that oxidative stress-related gene sets were up-regulated in OCCC, of which HNF-1β expression was observed in 95% of cases, which is consistent with the increased frequency of endometriosis in OCCC patients [41, 171]. These findings suggested that HNF-1β may be associated with severe oxidative stress in the OCCC tumor microenvironment. In addition, Liu et al. also found that the knockdown of HNF-1β in OCCC cells significantly increased cisplatin- or paclitaxel-mediated cytotoxicity [173]. The mechanism might be related to the fact that HNF-1β can protect cancer cells by regulating the glucose metabolism of OCCC cells and reducing ROS in the tumor microenvironment [145]. Our hypothesis is further supported by the findings of Lengyel et al., who successfully used metformin to modulate cellular metabolism in OC mice, thereby increasing cancer cells' sensitivity to platinum-based drugs [174]. Hence, we believe that HNF-1β inhibitors may be a future therapeutic strategy to improve the prognosis of OCCC patients, and the energy metabolism of tumor cells might direct us to future OCCC targeting research (Fig. 4C).

### Ferroptosis

Typically, tumor cells have a high metabolic activity as well as the ability to proliferate rapidly, which makes them more demanding of iron. Ferroptosis is a newly discovered mode of cell death. Unlike traditional apoptosis, necrosis, and autophagy, ferroptosis is due to excess intracellular iron ions, leading to oxidative stress and cell death, and has been linked to the development of many cancers, including OC [175, 176]. MEX3A is an evolutionarily conserved RNA binding protein (RBP) involved in cellular ferroptosis and is involved in various tumorigenesis by regulating mRNA stability, transport and translocation [177–179]. On the one hand, MEX3A can affect the distribution and utilization of intracellular iron ions by inhibiting the expression of iron metabolism-related genes FTH1 and FPN and regulating intracellular iron ion transport and storage, thus reducing the level of intracellular free iron ions and alleviating oxidative stress and cell death; On the other hand, MEX3A can also cause p53 protein degradation and enhance tumorigenesis [179]. In addition, Zou et al. found that OCCC has intrinsic vulnerability to ferroptosis by using small molecule drugs to inhibit GPX4, one of the key genes for cellular ferroptosis. They revealed that this intrinsic vulnerability is due to enhanced lipid peroxidation and reduced lipid synthesis after HIF pathway activation in tumor cells [180]. Endometriosis-derived mesenchymal stem cells (enMSC), characterized by loss of CD10 expression, can also promote the proliferation and invasion of OCCC cells by modulating the expression of iron metabolism-related genes, such as heparin-binding protein and transferrin and altering the intracellular iron ion content and distribution; Proliferation and invasion of OCCC cells also confer sensitivity to ferroptosis-inducing therapy [181]. The above results suggest that ferroptosis-related genes may be potential targets for effectively treating OCCC. The above results suggest that intracellular iron ions can be involved in tumor development in various ways, and interfering with iron metabolism in tumor cells to inhibit tumor development and metastasis through various modalities such as regulating the expression levels of ferroptosis-related genes and reversing tumor cell resistance to antitumor drugs seems to be a potential effective treatment for OCCC. However, the specific application of iron death in tumor therapy requires further research and exploration (Fig. 4D).

### Exploiting ARID1A synthetic lethal interactions

ARID1A mutations are found in 40–57% of OCCC patients, making it one of the most frequently mutated genes [182]. ARID1A plays a role in DNA double-strand brink (DSB) repair and the composition of Brahma-related gene 1/brahma (BRG1/ BRM)-associated factor (cBAF) complex and SWItch/Sucrose Non-Fermentable (SWI/SNF) complexes; therefore ARID1A mutation is considered as an early event of malignant tumor progression in OCCC [183]. Synthetic lethal means that two genes mutated in a single cell can interact with each other and result in different causes of cell death [184]. An increasing number of researchers have recently designed and implemented novel therapeutic strategies for various tumors, including OCCC, based on this mechanism.

Low recruitment of topoisomerase 2A (TOP2A) to chromosomes in ARID1A-deficient cells leads to decatenation defects during DNA replication and increased reliance on the cell cycle checkpoints enforced by Ataxia telangiectasia and Rad3-related (ATR) kinases [43]. ATR-inhibitor (VX-970) was found to interact with ARID1A synthetic lethal to kill ARID1A-deficient OCCC cells in both in vivo and in vitro studies [43]. Furthermore, ART inhibitors can disrupt DNA repair in tumor cells, releasing more tumor cell antibodies, as mentioned above [185]. Therefore, we conjecture that ICB therapy combined with ART inhibitor may be an effective treatment strategy for ARID1A-deficient cancer.

ARID1A is involved in the DSB process in tumor cells, similar to the role BRCA plays in tumor cells. The PARP-inhibitor has been widely recognized as an effective treatment for patients with BRCA mutated various tumors. As a result, we hypothesized that a PARP inhibitor could be used to treat patients with ARID1A-mutation tumors. This conjecture was confirmed in a 2015 trial in which Shen et al. found that ARID1A deficiency sensitizes cancer cells to PARP inhibitors in vitro and in vivo, providing a potential therapeutic strategy for patients with ARID1A -mutant tumors [186]. In addition, previous studies have found synergistic effects between ATR and PARP inhibitors in ARID1A-loss cells. Specifically, ATR inhibitors significantly increased the sensitivity of cells to PARP inhibitor treatment. In turn, the addition of PARP inhibitors to ARID1A-loss tumor cells and the addition of PARP inhibitors to the treatment of ARID1A-loss tumor cells could improve the responsiveness of cells to ATR inhibitors [187]. In this regard, we believe that this may be related to the fact that ATR is also involved in repairing damage to cellular DNA. A trial to compare the efficacy of combined ATR and PARP inhibitors in treating patients with ARID1A-loss or not OCCC is underway, and the results will be published soon (ATARI trial) [45].

Wu et al. found that ARID1A has a synthetic lethal interaction with aurora kinase A (AURKA) in colorectal cancer cells [188]. Specifically, AURKA inhibitors can cause more ARID1A-deficient cells to enter G2/M arrest and trigger tumor cell apoptosis because the loss of ARID1A can upregulate the expression level of AURKA, which acts as an oncogene that can cause tumor cell overriding cell cycle checkpoints by phosphorylate CDC25 Cat Ser198 via PLK1 and activate CDC25C nuclear translocation leading to tumor cells overriding cell cycle checkpoints, and enhancing cell proliferation, and suppressing apoptotic pathways [188, 189]. The same idea was expected for the treatment of ARID1A-loss OCCC patients. In a study of the Princess Margaret Phase II Consortium, researchers found that the application of ENMD-2076 (an AURKA inhibitor) in ARID1A-loss OCCC patients significantly prolonged the 6- month PFS rate (33% vs. 12%; *P* = 0.023) [46]. However, since ENMD-2076 also acts on VEGF, it is impossible to determine whether the benefit of ENMD-2076 is due to blocking AURKA. Therefore, clinical trials of drugs that specifically target the action of AURKA in OCCC patients are still required.

Miller et al. used dasatinib to treat OCCC and found that ARID1A-deficient OCCC cells had higher drug sensitivity to Dasatinib (a tyrosine kinase inhibitor, TKI), suggesting a potential synergistic lethal interaction between dasatinib and ARID1A [190]. Our speculation on the mechanism of action is as follows: On the one hand, TKI may act on P21 and Rb to cause more ARID1A-loss cells to enter the G1-S cell cycle arrest [190, 191]; On the other hand, some TKIs can inhibit the growth and division of cancer cells by inhibiting their angiogenesis, thereby reducing their nutrient and oxygen supply [192]. A single-arm phase II clinical study based on dasatinib showed limited efficacy of dasatinib in patients with ARID1A-mutant OCCC and endometrial CCC, with a RR of 3.8% (1/28) and mean PFS of 2.14 months [47]. Previous studies found that the sensitivity of the ARID1A-mutant OCCC cell lines to dasatinib was correlated with YES1 [190]. Therefore, we speculate that combining YES1 inhibitors and dasatinib may benefit ARID1A-mutant OCCC patients. However, no relevant studies have been published.

Polycomb Repressive Complex 2 (PCR2) is vital in regulating gene expression. It modifies histone H3 on chromatin and wraps it tightly in nucleosomes, making chromatin more compact and suppressing gene transcription and expression [193]. While Enhancer of zeste homolog 2 (EZH2), a key component of PCR2, can regulate ARID1A expression via acetylation modification, normal ARID1A expression can also limit EZH2's biological function [194, 195]. Bitler and colleagues used an EZH2 inhibitor (GSK126) to treat ARID1A-loss OCCC cells and found that ARID1A mutation status correlated with the response of tumor cells to EZH2 inhibitors [48]. The specific mechanism may be because the inhibition of EZH2 can lead to the upregulation of PIK3IP1 expression, which in turn inhibits PI3K-AKT signaling and contributes to the synthetic lethal interaction between ARID1A and EZH2 [196, 197]. Several clinical trials based on EZH2 inhibitors are underway, and their results will be published in the near future. Similarly, blocking the expression of histone deacetylase 2 (HDAC2), a component of PRC2, promotes histone deacetylation modifications that enhance histone stability in the nucleus, upregulating the expression of genes such as transcription factors E2F3 and TP73, thereby promoting tumor cell proliferation and inhibiting apoptosis [31, 198, 199]. Several studies have demonstrated significantly increased expression levels of HDAC2 in ARID1A mutant ovarian clear cell carcinoma [31]. Fukumoto et al. utilized pan-HDAC inhibitor (SAHA) to treat OC mice and found that ARID1A-mutant mice were more responsive to the drug and had longer survival than ARID1A-wild type mice, further supporting the above idea [49].

In addition, the researchers found that the co-occur rate of ARID1A and PI3KCA mutations was extremely high, and OCCC patients with PIK3CA mutations showed intense phosphorylated AKT immunoreactivity [200, 201]. These results suggest that PI3KCA mutations can promote tumor cell proliferation and metastasis by activating the PI3K-AKT-mTOR pathway. On the other hand, inactivated ARID1A may increase the mutation rate of PIK3CA by affecting the activity of the PIK3CA-AKT signaling pathway [202, 203]. In summary, PI3KCA inhibitors might inhibit the proliferation and invasion of ARID1A-deficient OCCC.

Glutathione (GSH), as a major intracellular antioxidant, protects tumor cells from reactive oxygen species (ROS) attack, while abnormal GSH metabolism is considered to be one of the characteristics of ARID1A-deficient OCCC cells [50, 204]. ARID1A mutations can lead to decreased expression of SLC7A11, which plays a vital role in encoding the XCT, a subunit of the cysteine/glutamate transporter, through multiple pathways [205]. It includes the inactivation of upstream transcription factors, altered Histone modifications, and DNA methylation [206, 207]. Thus, ARID1A-loss tumor cells must rely on the glutamate-cysteine ligase synthetase catalytic subunit (GCLC), a rate-limiting enzyme for GSH synthesis, to produce cysteine necessary for GSH production [50]. As a result, inhibition of GCLC activity could be a potential therapeutic strategy by exposing more tumor cells to ROS attack. Ogiwara et al. showed that GCLC inhibitors (APR246 and PRIMA-1) could markedly decrease GSH in ARID1A-deficient cancer cells, leading to apoptotic cell death triggered by excessive amounts of ROS [50]. In addition, other drug targets aimed at exploiting ARID1A synthetic lethal interactions, such as the heat shock factor 1 (HSF-1) pathway [51] and bromodomain and extra terminal (BET) [52], are being investigated (Fig. 4E).

### Inhibitors of downstream pathways of receptor tyrosine kinases and tyrosine kinases

Inhibitors of downstream pathways of receptor tyrosine kinases and tyrosine kinase can play an important role in tumor therapy by interfering with key processes of tumor cell proliferation, survival and metastasis. For example, Sunitinib, a tyrosine kinase inhibitor, has shown promising results in treating renal cell carcinoma and pancreatic neuroendocrine tumors [208]. Considering the similarity of gene expression profiles between OCCC and renal clear cell carcinoma, sunitinib has shown modest benefit for treating EOC [209, 210]. The phase II evaluation of sunitinib in treating persistent or recurrent clear cell ovarian carcinoma (GOG-254) showed that sunitinib had a median PFS of 2.7 months and median OS of 12.8 months in patients with recurrent or persistent OCCC. However, only two patients with OCCC (2/35) demonstrated PR or complete response (CR), suggesting that sunitinib demonstrated minimal activity in the second-and third-line treatment of persistent or recurrent clear cell ovarian carcinoma [211]. In addition, NRG-GY001 results showed that cabozantinib (targeting MET, VEGFR2, and RET) was used to treat 13 patients with recurrent OCCC with a median PFS of 3.6 months and median OS of 8.1 months and only 3 (23%) of these had a PFS ≥ 6 months [212]. Clinical trials using other drugs targeting tyrosine kinases for treating OCCC are ongoing, and the results will be published soon.

### Other potential therapeutic targets

ADP-ribosylation factor-like protein 4C (ARL4C) belongs to the family of small GTP-binding proteins 4 (small G proteins) and is a potential target for the treatment of OCCC. It is widely expressed in various human bodies, including ovarian follicular cells, and is involved in the proliferation and migration of various cancers [213, 214]. The study by Wakinoue et al. included 61 EM-associated ovarian cancer (EAOC), including 41 OCCC patients. The prognosis of EAOC patients with high ARL4C expression was found to be worse by statistical analysis (*p* = 0.036), and the expression of ARL4C was an independent risk factor affecting the prognosis of EAOC patients (5-year OS: HR = 12.048, *p* = 0.0201; 5-year PFS: HR = 8.130, *p* = 0.0036). Statistical analysis revealed that EAOC patients with high ARL4C expression have a worse prognosis (*p* = 0.036), and ARL4C expression was an independent risk factor affecting the prognosis of EAOC patients (5-year OS: HR = 12.048, *p* = 0.0201; 5-year PFS: HR = 8.130, *p* = 0.0036) [215].

In a study exploring the differential role of the Wip1-p38-p53 DNA damage response pathway in early/late-stage OCCC, researchers found that activation of the Wip1-p38-p53 pathway induced cell cycle arrest and apoptosis in early-stage tumors but promoted cell survival and proliferation in late-stage tumors [216]. Therefore, the effect of targeting the Wip1-p38-p53 pathway for OCCC may vary depending on the tumor stage.

Nagappan et al. found that the Caveolin-1-ACE2 axis in OCCC can regulate cellular metabolism and clearance of drugs, thereby affecting cellular sensitivity to cisplatin treatment [217]. The mechanism may be because Cav-1 can counteract the expression of Angiotensin-converting enzyme 2 (ACE2). The latter could limit the aryl hydrocarbon receptor (AhR) expression and the subsequent transcription of drug-metabolizing enzymes [218, 219]. Furthermore, the Cytochrome P450 3A4 (CYP3A4) enzyme is primarily found in tissues such as the liver and intestine, where it converts a variety of drugs, including cisplatin, into more easily excreted metabolites via oxidation and demethylation reactions, thereby influencing drug efficacy and toxicity [220]. Several studies have shown that ACE2 can upregulate CYP3A4 expression, suggesting its potential therapeutic role for OCCC [221, 222].

## Prognostic factors

### Tumor stage

Several studies have shown that tumor stage is an independent risk factor for the prognostic outcome of OCCC and that OS and PFS of OCCC patients decrease with increasing FIGO stage. The 3-year survival rates for OCCC at stages I, II, III, and IV are 80%, 47%, 34%, and 30% for PFS, and 96%, 85%, 54%, and 40% for OS, respectively [223, 224]. In addition, a meta-analysis based on 12 prospective Gynecologic Oncology Group (GOG) trials showed that early OCCC had a longer PFS than early HGSOC (HR 0.69, 95% CI 0.50 to 0.96), although there was no significant difference in OS (HR 0.76, 95% CI 0.53 to 1.09) [225]. A meta-analysis including 12 random control trials also showed no significant difference in OS (HR 0.87, 95% CI 0.75 to 1.02) [226]. However, several studies show that advanced-stage OCCC and recurrence of OCCC have a significantly worse prognosis than advanced-stage HGSOC [226, 227]. In addition, the prognostic outcomes of patients with distant organ metastases, including liver metastases, lung metastases, brain metastases, and PFS (26.7%), were significantly worse than those without organ metastases (5-year OS: 22.2% vs. 71.8%; 5-year PFS: 26.7% vs. 73.9%) [228]. In a study based on 170 OCCC patients from China, combined ascites were shown to be an independent risk factor for OCCC patients (HR: 2.354; 95% CI: 1.118–4.762), with a lower 2-year PFS rate in patients compared to those without ascites (87.1% vs. 49.9%) [229].

### Treatment factors

The extent of cytoreduction is an independent risk factor for the prognosis of OCCC patients. A GOG study found that OCCC patients with small-volume disease had worse prognostic outcomes [110]. Bai and colleagues showed that patients with RD > 1 cm had worse OS than those with RD < 1 cm (HR = 2.48, 95% CI = 1.46–2.41) [230]. Similarly, the findings of Yano et al. further support that ideal tumor reduction significantly prolongs OS in patients with OCCC (HR = 17.2; 95% CI = 6.90–43.5) [5]. However, no study has explored the relationship between the degree of residual postoperative lesions and PFS. Because most OCCC patients are combined with EMs, heavy pelvic adhesions develop, making tumor dissection more difficult and increasing the risk of intraoperative tumor rupture. Rupture of OCCC may lead to a medically induced increase in the tumor stage. However, it is unclear whether tumor rupture affects the prognosis of patients with OCCC. In a study of 241 patients with OCCC I stage, Hoskins et al. found that 5-year disease-free survival (DFS) was better in patients with stage IC with negative cytology than stage IA/IB (92% vs. 84%), whereas patients with unknown/positive cytology and/or unknown/positive surface involvement had a worse prognosis, with a 5-year DFS of 48% [231]. A study by Mizuno et al. found that patients with negative cytology had significantly better 5-year DFS than those with positive cytology (86% vs. 41%) [121]. However, a Korean study found no significant difference in 5-year PFS (88.8% vs. 91.7%; *p* = 0.291) and 5-year OS (94.6% vs. 95.4%; *p* = 0.444) between patients with stage IC1 OCCC and stage IA/IB OCCC. A study by Hoskins et al. also concluded that stage IC2/IC3 was the only independent poor prognostic factor for OS (HR, 3.50; 95% CI: 1.31 to 9.36) [227]. In conclusion, we believe that peritoneal cytology is closely related to the prognosis of OCCC patients, and careful postoperative manipulation can possibly avoid medically induced tumor rupture.

The sensitivity of OCCC patients to platinum, including that of other EOC subtypes, is an important factor influencing prognosis. A retrospective data including 75 OCCC patients showed platinum-sensitive patients had longer survival than platinum-resistant patients (16 months vs. 7 months) [140]. However, there was no significant difference in the overall survival of patients receiving non-platinum-based chemotherapy compared to those receiving standard chemotherapy regimens [6, 232].

### Status of lymph nodes

The status of regional LNs retrieved during surgery is not only an independent prognostic factor but also an essential factor in assessing the risk of recurrence of patients with EOC [233]. The American Joint Committee on Cancer/International Union Against Cancer (AJCC/UICC) tumor-node-metastasis (TNM) classification is widely used to predict prognosis. However, because it is calculated solely on the absolute number of positive LNs, it may result in an underestimation of the N-stage. Therefore, many novel LNs staging systems have been proposed to improve the assessment of prognosis in EOC. Based on several studies, the LNR (the ratio of PLNs/RLNs) has been considered a novel LN system and has been proven to be associated with unfavorable prognosis in OCCC patients [234, 235]. Log odds of positive lymph nodes (LODDS), which is calculated with the following expression: log (PLNs + 0.5) / (RLNs-PLNs + 0.5), comprehensively considers the effect of the number of positive lymph nodes (PLNs) and resected lymph nodes (RLNs) on the prognosis for tumor patients and has been widely proven as an effective prognosis prediction tool and a novel lymph node staging system in OCCC [228, 236].

LVSI not only affects the prognostic outcome of early-stage OCCC patients but also is associated with tumor recurrence and death. Positive LVSI patients had a 5-year DFS and 5-year OS of 59.0% and 81.2%, respectively, compared to 85.5% and 95.9% in patients with negative LVSI [237, 238].

### Recurrent disease

To our knowledge, no published guidelines exist for effectively treating relapsed OCCC, resulting in a higher recurrence rate and worse prognosis for OCCC compared to other EOC subtypes. Kajiyama et al. reported that the relapse rate of OCCC was stage-related, with stage I-IV relapse rates of 29%, 30%, 62%, and 73%, respectively, and that patients who relapsed tended to have lower 5-year survival (13.2%) and shorter a post-recurrence survival time (10.0 months) [239]. We believe this may be related to the lower RR to chemotherapy in patients with OCCC. The RR to second-line regimens is only 1–10%, and the RR for patients with platinum resistance tends to be even lower [6, 41]. Crotzer et al. showed that only 9% of platinum-sensitivity showed PR to treatment with PTX plus carboplatin, while only 1% of platinum-resistance patients showed PR [139].

### Other factors

Although CA125 is not very specific and sensitive for the diagnosis of OCCC, it is a useful prognostic predictor and seems to respond to some extent to the responsiveness of OCCC patients to chemotherapy drugs. Bai and colleagues found that the time to normalization of CA125 in the second cycle, between the second and the sixth cycle, and never normalized, 5-year OS were 94.0%, 95.2%, 50.1%, and 36.7% year OS was 94.0%, 95.2%, 50.1%, and 36.7%, respectively [230]. However, there is still a lack of published studies exploring the relationship between CA125 and drug resistance in patients with OCCC.

VTE is more common in advanced OCCC cases and more frequent than other histologic types of OC [240]. In addition, VTE is also considered an independent risk factor for OCCC as it increases the risk of various adverse events, including cardiovascular accidents and postoperative complications during the OCCC treatment [241]. Several studies found shorter OS and PFS in patients with combined VTE, which persisted after correction for other clinical factors [242, 243]. Meanwhile, the tumor recurrence and death rates were significantly higher in patients with combined VTE [244]. Therefore, we believe that patients with OCCC should be vigilant for VTE during treatment and that appropriate thromboprophylaxis, such as low molecular heparin, is required. Some drugs that include targeting coagulation factors [245], anti-inflammatory factors [246], and HIF1A/VEGF/VEGFR pathway inhibitors [247] are effective and deserve further promotion.

Although the prevalence of EMs in EOC has been calculated to be 4.5%, 1.4%, 35.9%, and 19.0% for serous, mucinous, clear-cell, and endometrioid OC, respectively, the impact of EMs on the prognosis of OCCC patients is controversial, as previously described. OCCC patients with a history of existing EMs tend to be diagnosed earlier due to the earlier onset of clinical symptoms [248]. They have lower positive LVSI (23% vs. 51%) [237], lower LNM rate (17%-41%) [238], and better survival time (5-year OS. 70.2%—74.1%). However, other studies found no statistically significant prognostic impact of EMs on OCCC after controlling other clinical factors [249, 250].

In a SEER-based study, Chen et al. found that despite a smaller proportion of patients with bilateral OCCC (10.8%), prognostic outcomes tended to be worse than in patients with unilateral disease (HR: 1.789; 95% CI: 1.283–2.496) [228]. This was further supported by the findings of Wang and colleagues (HR: 1.097; 95% CI: 1.006–1.195) [236]. In addition, several studies have shown that age and tumor size are independent risk factors affecting the prognosis of OCCC patients [228, 229].

## Conclusion

In summary, Ovarian Clear Cell Cancer (OCCC) is a complex and aggressive disease that poses significant treatment challenges. With distinct clinical presentation, molecular features, and biological behavior compared to other EOC histological subtypes, OCCC requires tailored multidisciplinary care to achieve the best possible outcome. While cytoreductive surgery and chemotherapy are the mainstay of therapy, the survival rate of patients with advantaged- stage OCCC remains poor due to drug resistance, recurrence, and metastasis. To address these challenges, several targeted therapies and immunotherapies have recently entered clinical trials with promising results. However, more research is necessary to elucidate specific pathogenic and drug resistance mechanisms and to develop accurate predictive biomarkers for more individualized and precise treatment of OCCC patients. Close international cooperation in conducting clinical trials through academic research groups is needed to explore more effective treatment options. In addition, more accurate predictive biomarkers are required to provide more precise and individualized treatment for each OCCC patient.

## Data Availability

The data supporting the conclusions of this article are included within the article and are available from the corresponding author on reasonable request.

## Abbreviations

EOC: Epithelial ovarian cancer
OC: Ovarian cancers
WHO: The World Health Organization
OCCC: Ovarian clear cell cancer
SOC: Serous ovarian cancer
Ems: Endometriosis
IOTA: The international ovarian tumor analysis
GOG: Gynecologic Oncology Group
SIR: Systemic inflammatory response
LMR: Lymphocyte-to-macrophage ratio
HNF1-β: Hepatocyte nuclear factor-1-beta
ABCB1: Adenosine triphosphate-binding cassette subfamily B member 1
ABCC1: Adenosine triphosphate-binding cassette subfamily C member 1
ABCC3: Adenosine triphosphate-binding cassette subfamily C member 3
ABCF2: Adenosine triphosphate-binding cassette subfamily transporter F2
ABC: Adenosine triphosphate-binding cassette
PTX: Paclitaxel
OSAC: Ovarian serous adenocarcinoma
GPx3: Glutathione peroxidase 3
GLRX: Glutaredoxin
SOD2: Superoxide dismutase
ERCC1: Excision repair cross-complementation group 1
XPB: Xeroderma pigmentosum group B
MMR: Mismatch repair systems
MSI: Microsatellite instability
EGFR: Epidermal growth-factor receptor
HER2: V-erb-b2 erythroblastic leukemia viral oncogene homolog 2
PI3K: Phosphatidylinositol 3′-kinase
BAD: Phosphorylated Bc1-2 antagonist of cell death
Bcl-2: B-cell leukemia/ lymphoma
FGFR3: Fibroblast growth factor receptor 3
CDKs: Cyclin-dependent kinases
NCCN: National Comprehensive Cancer Network
FIGO: Federation International of Gynecology and Obstetrics
ESMO-ESGO: European Society for Medical Oncology-European Society of Gynaecological Oncology
GCIG: Gynecologic Cancer InterGroup
PFS: Progress Free Survival
RLNs: Resection lymph nodes
LNs: Lymph nodes
OS: Overall survival
FSS: Fertility-sparing surgery
NCDB: The National Cancer Database
DSS: Disease specific survival
CSS: Cancer specific survival
SAR: Survival after recurrence
NACT: Neoadjuvant chemotherapy
PDS: Primary cytoreductive surgery
HR: The hazard ratio
ICB: Immune checkpoint blockade
PR: Partial response
MLH1: MutL hmolog 1
MSH2: MutS homolog 2
MSH6: MutS homolog 6
PMS2: Postmeiotic segregation increased 2
TMB: Tumor mutational burden
IDO: The indoleamine-pyrrole 2,3-dioxygenase
GPC3: Glypcan-3
RR: Response rate
DCR: Disease control rate
BRG1/ BRM: Brahma-related gene 1/brahma
cBAF: Brahma-related gene 1/brahma-associated factor
SWI/SNF: SWItch/Sucrose Non-Fermentable
TOP2A: Topoisomerase 2A
ATR: Ataxia telangiectasia and Rad3-related
DSB: Double-strand brink
AURKA: Aurora kinase A
EZH2: Enhancer of zeste homolog 2
H3K27me3: Trimethylation of Lys-27 in histone 3
PCR2: Polycomb Repressive Complex 2
HDAC2: Histone deacetylase 2
GSH: Glutathione
ROS: Reactive Oxygen Species
GCLC: The glutamate-cysteine ligase synthetase catalytic subunit
HSF-1: Heat shock factor 1
BET: Bromodomain and extra terminal
HNF-1β: Hepatocyte nuclear factor‑1β
RBP: RNA-binding proteins
enMSC: Endometriosis-derived mesenchymal stem cells
ARL4C: ADP-ribosylation factor-like protein 4C
EAOC: EMs-associated ovarian cancer
ACE2: Angiotensin-converting enzyme 2
AhR: Aryl hydrocarbon receptor
GOG: Gynecologic Oncology Group
DFS: Disease-free survival
TKI: Tyrosine kinase inhibitors
